# Mental imagery interventions reduce subsequent food intake only when self-regulatory resources are available

**DOI:** 10.3389/fpsyg.2014.01391

**Published:** 2014-11-28

**Authors:** Benjamin Missbach, Arnd Florack, Lukas Weissmann, Jürgen König

**Affiliations:** ^1^Department of Nutritional Sciences, University of Vienna, ViennaAustria; ^2^Department of Psychology, University of Vienna, ViennaAustria

**Keywords:** mental imagery, self-regulation, satiation, habituation, depletion, regulation of food intake

## Abstract

Research has shown that imagining food consumption leads to food-specific habituation effects. In the present research, we replicated these effects and further examined whether the depletion of self-regulatory resources would reduce the habituation effects of imagined food consumption. Since self-regulatory resources have been shown to reduce habituation effects during the perception of emotional stimuli, we expected a reduction in habituation effects from imagined food consumption when self-regulatory resources were depleted. In Study 1, we replicated habituation effects as a response to imagining gummy bear consumption with a high (36) and medium number (18) of repetitions in a camouflaged taste test. Participants imagining gummy bear intake showed decreased food intake compared with participants who imagined putting a coin into a laundry machine. The number of repetitions did not significantly moderate the observed habituation effect. In Study 2, we investigated whether self-regulatory depletion would impede habituation effects evoked by the imagination of walnut consumption. Participants in a depleted state did not show a reduction in food intake after imagining walnut intake compared with participants in a non-depleted state. We discuss directions for future research and processes that might underlie the observed moderating effect of self-regulatory resources.

## INTRODUCTION

Food intake is important for survival but also has negative consequences such as overweight and obesity, which are accompanied by massive societal and financial burdens (Reilly and Kelly, 2011). Thus, understanding the fundamental underlying mechanisms of food intake is pivotal. A fact challenging individuals’ self-regulation is the presence of food cues in their environments (Swinburn et al., 1999). Indeed, a common view is that cues leading individuals to imagine the consumption of food (e.g., cues from sensory marketing) powerfully increase appetite and the likelihood of food consumption through perceptual modulation (Barsalou, 2008; Elder and Krishna, 2010). However, although this might be true in many cases, researchers have shown that mechanisms of habituation can help individuals cope with these challenges (Papies et al., 2012). Research on sensory-specific satiety, for instance, has shown that individuals habituate to specific foods while consuming such foods (Rolls et al., 1981; Hetherington, 1996) in a modality-specific way (Havermans and Mallach, 2014). Even more interesting, recent research has also found that individuals habituate while imagining the consumption of specific food (Morewedge et al., 2010). Since research on the habituation effects of imagined food consumption is rare, the objective of the present research was to examine whether these kinds of habituation effects could be replicated across different kinds of food items, different amounts of repetitions and most importantly whether similar limiting circumstances would hold for imagined food consumption as they do for other kinds of habituation. In particular, we studied whether habituation effects would decrease when self-regulatory resources were depleted compared with when they were not depleted. This latter point is of major relevance because the depletion of self-regulatory resources usually increases impulsive behavior such as the consumption of palatable food (Vohs and Heatherton, 2000; Hagger et al., 2010; Heatherton and Wagner, 2011), and it would be of interest to determine whether reduced habituation effects contribute to such phenomena.

Repeatedly imagining oneself eating a certain food was shown to decrease subsequent consumption of the same food (Morewedge et al., 2010). To further contribute to the understanding of such habituation effects, we investigated whether the effects would increase with the number of repetitions, whether they would occur for different food items, and whether they would be reduced when self-regulatory resources were depleted.

### THEORETICAL BACKGROUND

Perception and food cognitions involve perceptual simulations and mental images (Bensafi et al., 2007; Barsalou, 2008). Thinking about a desired food increases a person’s motivation to consume that food (elaboration intrusion theory of desire) and is defined as an interplay between associative and elaborative cognitive processes (Kavanagh et al., 2005). External food cues trigger the activation of anticipatory signaling of the autonomous nervous system in preprandial phases. For example, external food cues increase the production of gastric juices and saliva and activate hormonal release to prepare the body for impending food intake (Rodin, 1985). At the same time, the desire to eat increases (Dadds et al., 1997). Spontaneous mental images can lead to similar responses (May et al., 2012). Such images are involved in the development of cravings for alcohol (Statham et al., 2011) and food (Tiggemann et al., 2010; Kemps et al., 2012; Meule et al., 2012; Bullins et al., 2013; Rodriguez-Martin et al., 2013).

Whereas it is evident that thoughts about food can enhance appetite, recent research also suggests that repeatedly imagining food consumption upon being instructed to do so by an experimenter may lead to different effects than thoughts about food without multiple repetitions. In a landmark study Morewedge et al. (2010) showed that 30 repetitions of imagining the consumption of a specific food (e.g., M&M’s; cheese cubes) resulted in decreased consumption of the food afterward compared with three repetitions. Hence, imagining food intake does not always lead to increases in food intake. Morewedge et al. (2010) speculated that habituation processes are involved in these effects. Indeed, habituation effects for food consumption have been found for the actual consumption of food before (Epstein et al., 2009a,b; Carr and Epstein, 2011).

According to the memory-based associative conditioning theory, habituation occurs when the presentation of new stimuli is no longer surprising. The presentation of stimuli that already represent information that is stored in short-term memory leads to a reduction in stimulus response (Wagner et al., 1981; Epstein et al., 2009b). A core principle of the standard operating procedure (SOP) model is that when a stimulus is presented, the stimulus is represented in the form of a memory node, which is then activated to a high state of activity (maximally active; the A1 state). Over time, the activity decays, leading to a lower level of activation (processing is more peripheral; the A2 state), and after further decay, such activity becomes inactive (the I state). Information flow is unidirectional from the A1 to A2 to I states; thus, processing in the other direction (from A2 to A1) cannot occur. During food intake, a switch between a state of maximum activity (A1) to a less active and more peripheral processing state (A2) occurs. Sensory-specific satiety, a decrease in the ability to derive pleasure from a certain food after repeatedly being exposed to it, is therefore defined as a habituation process (Rolls et al., 1981; Swithers and Hall, 1994). Repeatedly thinking about consuming a certain food can be seen as a simulation of real food intake without being physically exposed to the food. The experience is similar to an exposure to internally stored memories about the sensory, contextual, and emotional characteristics of an experience (Pylyshyn, 2002; Kosslyn, 2003). When objects are visualized, neural regions are activated in a way that is similar to the actual process of seeing the objects (Ganis et al., 2004), although qualitative differences in the neural dynamics can be observed (Lee et al., 2012; Johnson and Johnson, 2014). Hence, simulating food intake appears to evoke regulating mechanisms that are similar to those evoked from actual food intake.

Interestingly, the SOP model implies (Wagner et al., 1981), that memory processes are the basis for habituation processes and that habituation should be reduced when these memory processes are blocked. In line with this assumption, research has found that distraction is able to reduce habituation effects. For example, individuals were less likely to habituate to the consumption of popcorn when being distracted by actively watching a movie (Epstein et al., 2009b; Harris et al., 2009). Also, results of a recent meta-analysis supported the assumption that distraction leads to an increase in the amount of food consumed (Robinson et al., 2013). A different line of research has shown that the depletion of self-regulatory resources blocks inhibition in eating behavior (Vohs and Heatherton, 2000; Kahan et al., 2003). This research did not study habituation effects directly, but it showed that individuals with reduced self-regulatory resources were less likely to limit their food intake. Self-regulation and executive functioning are proposed to share the same resources. Depleting these resources was associated with reduced inhibitory effects (Kaplan and Berman, 2010). Direct evidence for habituation-reducing effects of the depletion of self-regulatory resources comes from research on the perception of emotional stimuli. Wagner and Heatherton (2013) asked participants in one condition to complete a task that demanded self-regulatory control. In this task, participants had to watch a 7-min nature documentary and inhibit their reading of words presented at the bottom of the screen. They found that this task resulted in a reduction in habituation as a response to emotional pictures observed in the amygdala compared with a control task (Wagner and Heatherton, 2013). Since habituation needs cognitive resources to occur, and since self-regulatory depletion already impedes habituation on the level of very basic brain processes, we posit that habituation while imagining food intake should decrease when individuals’ self-regulatory resources are depleted.

### THE PRESENT STUDY

The main objectives of the present study were first to replicate the habituation effects after the repeated imagination of food consumption with different food items and different numbers of repetitions and second to test whether the effects would be moderated by self-regulatory depletion. To replicate (Morewedge et al., 2010) findings, we used gummy bears in Study 1 and walnuts in Study 2. To examine the moderating effect of self-regulatory depletion, we varied whether participants had to complete a depleting task in advance in Study 2.

According to the SOP model, increasing the number of repetitions should result in an even more pronounced effect on behavior – thus leading to a larger reduction in food intake. Therefore, we hypothesized that in conditions with a larger number of repetitions, the reduction in food intake due to habituation would be higher than in conditions with a smaller number of repetitions. Therefore, we varied the number of repetitions in Study 1 and tested for habituation in the different conditions.

We furthermore posit that the effects of repeatedly imagining food consumption are based on very general mechanisms and are not linked to a specific kind of food. Therefore, we introduce two new foods into the experimental paradigm to broaden the food spectrum. Morewedge et al. (2010) used foods containing large amounts of fat (cheese cubes: ∼20–30% fat content) and sugar (M&M’s: ∼66% sugar) with specific sensory and health characteristics. In the present research, we examined whether habituation would occur after participants imagined themselves consuming gummy bears (Study 1) and walnuts (Study 2). We used gummy bears to study whether the effect could be replicated with a food that is relatively easy to imagine (mignon design in the form of bears). We expected to observe habituation effects after participants repeatedly imagined the consumption of gummy bears. In Study 2, we used walnuts because they are only marginally processed foods and do not include added micro or macronutrients. Although walnuts contain a high amount of fat (∼63%), generally walnuts are regarded as healthy and natural foods. In contrast, M&M’s, cheese, and gummy bears are highly processed foods. Again, we argue that habituation is defined as a very general mechanism that occurs independently from the imagined food. We expected to observe habituation effects after participants imagined walnut consumption.

Finally, we argue that habituation to the target food should occur only when cognitive resources are available. If cognitive resources are intact, it should be possible to induce the habituation effect. That is, the switch from the A1 to the A2 state will occur, and food intake should be reduced. On the other hand, if cognitive resources are depleted, no habituation effect should occur, and food intake should not be reduced. Therefore, we hypothesized that when a person is in a state of self-regulatory depletion, cognitive resources are not available for habituation to occur. Thus, performing mental imagery with foods in this condition does not lead to habituation and no decrease in food intake in a subsequent taste test was expected (Study 2).

## STUDY 1

The main objectives of Study 1 were to replicate the habituation effect after participants imagined food consumption and to test this effect with a large and medium number of repetitions. To examine these objectives, we applied a 2 (*number of repetitions*: 18 vs. 36) × 2 (*imagery item*: gummy bears vs. coins) between-subjects design. Participants were asked to imagine consuming gummy bears or inserting a coin into a laundry machine. We expected a habituation effect after the imagined consumption of the gummy bears and that the habituation effect would be stronger for 36 compared with 18 repetitions.

### MATERIAL AND METHODS

#### Participants

A sample of 101 undergraduate students from the University of Vienna participated in the study on a voluntary basis. Participants were recruited via Internet forums, social media, and flyer postings on the campus of the University. They were asked to refrain from eating 3 h before the experiment and were blinded to the true intentions of the study. They believed they were taking part in a taste and rating test of gummy bears. All ranges of BMI and age were included in the sample. Six participants were excluded from the statistical analyses because they indicated that they did not imagine food consumption as they were asked to. This resulted in a total sample of 95 participants (77 female and 18 male) with a mean age of 24.01 years (SD = 5.1) and a mean BMI of 22.02 kg/m^2^ (SD = 2.7) across both sexes. Ten participants (four male, six female) were classified as overweight (BMI ≥ 25 kg/m^2^) and one male participant as obese (BMI ≥ 30 kg/m^2^), whereas all other participants displayed a BMI between 18.5 and 24.99 kg/m^2^ and were therefore considered to have a normal weight.

#### Study design

The study was designed as a camouflaged taste test of gummy bears presented immediately after a repetitive mental imagery paradigm. This intervention involved repeatedly imagining the consumption of gummy bears or, as a control, visualizing oneself putting coins into a laundry machine with a given number of repetitions of either 18 or 36. Participants were randomly assigned to the 2 (*number of repetitions*: 18 vs. 36) × 2 (*imagery item*: gummy bears vs. coins) between-subjects design using urn randomization (Wei and Lachin, 1988). Unbeknownst to the participants, the qualitative results of the subsequent taste test were not analyzed further, whereas in fact, the amount of gummy bears consumed was the main variable of interest in this study.

#### Procedure

After arriving at the laboratory, all participants stated their age, sex, body weight, and height and completed questionnaires on current hunger, fullness, overall liking of gummy bears, and restrained eating. Subsequently, participants were asked to perform the mental imagery paradigm, which was explained as a “test of mental visualization skills.”

Participants in the gummy bear condition received detailed instructions on how to imagine the consumption of gummy bears either 18 or 36 times. Participants in the control condition were told to imagine putting a 50¢ coin into a laundry machine (motor control task) with an equivalent number of repetitions. The control task was designed to involve imagined motor behaviors that were similar to those from the imagined consumption task. The numbers of 18 and 36 repetitions were chosen to balance between applied practicability and to avoid demanding too much or too little from the participants. A standard package of gummy bears available in an Austrian supermarket contains 72 gummy bears. Thus, 36 repetitions represents half of a standard package available at Austrian supermarkets. Followed by a short introduction from the experimenter, participants were seated in one of the eight separated booths of our sensory lab. They were given detailed written instructions on the mental imagery task procedure (see Table S1). In the description of the task, we stressed a precise wording and asked the participants in the consumption imagery group to focus on sensory and textural characteristics of the imagined food item and on the eating experience of the imagined food itself. In the control group, participants were asked to focus on the sensory and textural characteristics of the imagined coin item and on the action (motoric) experience of throwing the coin in the laundry machine. To keep track of each repetition, we asked the participants to count each repetition on a checklist using pen and paper. We verified the checklists at the end of the experiment. Additionally, we encouraged the participants to spend at least 15 s for every repetition, but did not measure the cumulative time spent for the complete mental imagery task.

After the imagination task, participants were asked to engage in a taste test of different colored gummy bears. Each bowl contained 83 g of gummy bears. Participants were told to eat *ad libitum* from the bowl. The bowls were weighed before and after the taste test to assess the amount of gummy bears eaten. Also, participants rated their hunger, fullness, and liking of the gummy bears once more, completed questionnaires to assess the vividness of their mental imagery, and answered the manipulation check question about whether or not they performed this task. Finally, participants were informed that the experiment was over, were debriefed, thanked for their cooperation, and dismissed.

### MEASURES

#### Manipulation check

To examine whether participants really performed the required mental imagery task, they were asked to indicate whether they had conducted the task or not (“Did you really perform the task we asked you to?”).

#### Hunger status and liking of the product

Visual analog scales (VASs) were used to measure appetite sensations (Stubbs et al., 2000). We used a 120 mm horizontal line with the extremes of the sensations *hunger* and *fullness* at the ends of the line. Participants had to quantify their subjective feeling by placing a mark across the line. We asked participants “how hungry do you feel?” and “how full do you feel?” with the anchor points “0 = not hungry” and “120 = very hungry” and “0 = not full” and “120 = very full,” respectively. To measure how much they liked the product, we used a VAS with three anchor points (“not at all,” “neither…nor,” “very much”).

#### Visual imagery

To assess visual imagery, participants were asked to visualize visual images and rated four different scenes on the vividness of four different aspects of these scenes on a 5-point Likert scale (1 = *no picture at all; you merely know that you are thinking about the object*; 5 = *perfectly clear; as vivid as normal vision*) using the Vividness of Visual Imagery Questionnaire (Marks, 1973). An everyday preference for using visual mental images was assessed via the Individual Difference Questionnaire consisting of 13 statements. Participants rated their agreement with each statement on the 5-point Likert scale (1 = *complete agreement*; 5 = *complete disagreement*). Adapted German versions of both questionnaires were used (Hirschfeld et al., 2012) with an internal consistency in the current study of α (Vividness of Visual Imagery Questionnaire) = 0.93 and α (Individual Difference Questionnaire) = 0.72.

#### Restrained eating

An adapted German version of the 10-item Restraint Scale (Dinkel et al., 2005) was used to assess concern for dieting and weight fluctuation among participants. Concern for dieting was assessed with questions about dieting frequency (0 = *never;* 4 = *always*), weight fluctuation affecting the participants’ lives (0 = *not at all*; 3 = *very much*), sensible eating in front of others (0 = *never*; 3 = *always*), thinking about food all the time (0 = *never;* 3 = *always*), feeling guilty after overeating (0 = *never*; 3 = *always*), and mindfulness of one’s own eating behavior (0 = *not at all*; 3 = *extremely*). Dieting frequency was assessed with questions regarding their maximum amount of weight gain in kilograms within 1 month (0 = *0–2.5 kg*; 1 = *2.5–5 kg*; 2 = *5–7.5 kg*; 3 *= 7.5–10 kg*; 4 = > 10 kg), their maximum amount of weight gain in kilograms within 1 week (0 = *0–0.5 kg*; 1 = *0.5–1 kg*; 2 = *1–1.5 kg*; 3 *= 1.5–2.5 kg*; 4 = > 2.5 kg), their typical weight fluctuation within 1 week (0 = *0–2.5 kg*; 1 = *2.5–5 kg*; 2 = *5–7.5 kg*; 3 *= 7.5–10 kg*; 4 = > 10 kg), and their maximum weight in kilograms above their desired weight in kilograms (0 = *0–0.5 kg*; 1 = *0.5–3 kg*; 2 = *3–5 kg*; 3 *= 5–10 kg*; 4 = > 10 kg). The scale values were averaged across the items. High values indicated restrained eating. Internal consistency in the current study was α (Restraint Scale) = 0.67.

#### Eating behavior

The amount of gummy bears consumed served as the primary dependent variable. We weighed the bowl of gummy bears before and after the experiment with a standard scale to three decimals places. At the end of the experiment, we assessed participants’ reasoning about the possible effect of imagining food intake on their hunger status.

#### Ethics statement

The experimental procedure was reviewed and approved by the University of Vienna Ethics Committee (*reference number*: 00065), and written informed consent was obtained from all participants before data collection. Participants were informed that they could withdraw their participation at any time during the experiment.

#### Statistical analysis

Results were considered significant at an α level of *p* ≤ 0.05. Results marked *ns* refer to *p*-values > 0.05 (for a summary of the data, see Table S2).

### RESULTS AND DISCUSSION

#### Eating behavior

**FIGURE 1:**
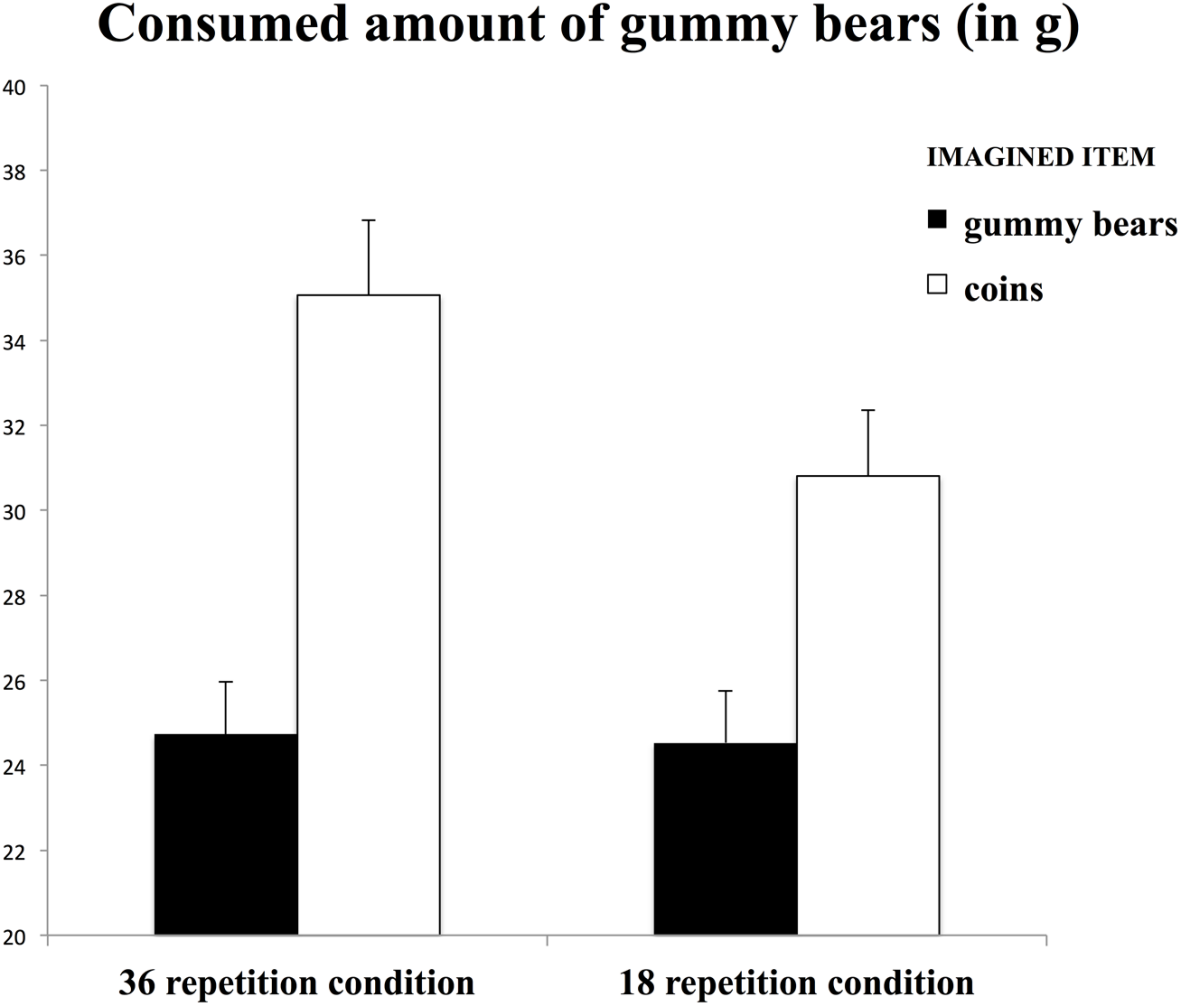
**Consumed amount of gummy bears (in **g**) as a function of imagined item (gummy bears vs. coins) and amount of repetitions (18 repetitions vs. 36 repetitions).** Error bars indicate standard errors of the means (Study 1).

We hypothesized that participants would eat a smaller amount of gummy bears when they repeatedly imagined eating gummy bears compared with putting coins into a laundry machine. Furthermore, we expected this effect to be more pronounced for 36 compared with 18 repetitions. To test the hypotheses, a 2 (*repetition number*: 18 vs. 36) × 2 (*imagery item*: gummy bears vs. coins) between-subjects ANOVA with gummy bear intake as the dependent variable was computed. As expected, participants consumed a smaller amount of gummy bears when they repeatedly imagined eating gummy bears (*M =* 24.63 g, SD = 11.02 g) compared with when they repeatedly imagined putting coins into a laundry machine (*M* = 32.94 g, SD = 16.94 g) [*F*_(3,95)_ = 8.61, *p* = 0.004, $\eta_{p}^{2}$ = 0.09] (see **Figure 1**). In contrast to our hypothesis, the interaction between repetition number and imagery item was not significant [*F*_(1,95)_ = 0.51, *p* = 0.47].

As expected, liking gummy bears had a pronounced effect on gummy bear consumption [*F*_(1,95)_ = 13.8, *p* = 0.04, $\eta_{p}^{2}$ = 0.13]. Liking gummy bears did not change significantly from before (*M* = 80.17, SD = 23.58) to after the experiment was conducted (*M* = 77.55, SD = 26.99) [*F*_(1,95)_ = 0.12, *p* = 0.29]. None of the following parameters had effects on the amount of gummy bears consumed: (i) hunger assessed before the experiment [*F*_(1,95)_ = 0.36, *p* = 0.55]; (ii) fullness before the experiment [*F*_(1,95)_ = 0.11, *p* = 0.78]; (iii) time of last meal intake [*F*_(1,95)_ = 0.14, *p* = 0.71]; (iv) restrained eating scores [*F*_(1,95)_ = 1.26, *p* = 0.27]; (v) BMI scores, [*F*_(1,95)_ = 0.39, *p* = 0.53]; (vi) gender [*F*_(1,95)_ = 2.80, *p* = 0.09]; (vii) Vividness of Visual Imagery Questionnaire scores [*F*_(1,95)_ = 0.08, *p* = 0.78]; or Individual Difference Questionnaire scores [*F*_(1,95)_ = 0.29, *p* = 0.59]. In an analysis of covariance, the inclusion of the above variables did not affect the reported habituation effect [*F*_(1,95)_ = 4.32, *p* = 0.04, $\eta_{p}^{2}$ = 0.05].

No changes in fullness from before (*M =* 49.32, SD = 26.91) to after the mental imagery task (*M =* 58.26, SD = 25.89), [*F*_(1,94)_ = 1.27,* p* = 0.29] or in hunger from before (*M* = 33.26, SD = 24.41) to after the mental imagery task (*M* = 28.99, SD = 23.69), [*F*_(1,94)_ = 0.81, *p* = 0.48], were observed.

#### Participants’ expectations

To examine whether the reported habituation effect could be evoked by participants’ expectations, we also analyzed these expectations. Importantly, most participants did not expect a habituation effect. 90% of the participants inferred that imagining gummy bear intake might stimulate their appetite, 8% reasoned that thinking about food intake might decrease their appetite, and 2% assumed that it might have no effect on their appetite. When we excluded participants who expected a decrease in their appetite (eight participants), a between-subjects ANOVA still showed a significant main effect of the imagery task [*F*_(3,87)_ = 7.51, *p* = 0.007, $\eta_{p}^{2}$ = 0.08], indicating a lower consumption of gummy bears among participants who repeatedly imagined eating gummy bears (*M* = 25.00 g, SD = 11.27 g) compared with those who repeatedly imagined putting coins into a laundry machine (*M* = 33.31 g, SD = 16.13 g).

To sum up, in Study 1, we replicated the habituation effect after imagining food consumption. Importantly, the participants’ expectations could not account for this effect.

Morewedge et al. (2010) found no habituation effect for three compared with 30 repetitions. Hence, at least more than three repetitions are necessary to produce the effect. In Study 1, the number of repetitions (18 vs. 36) did not significantly moderate the effect. We expected that a larger number of repetitions would be necessary for the habituation effect to occur. The present data did not confirm this hypothesis but reflected the strength of the habituation effect. In Study 2, we examined whether self-regulatory depletion would impede habituation effects while participants imagined food consumption.

## STUDY 2

The main objective of Study 2 was to test the hypothesis that a depletion of self-regulatory resources would decrease the habituation of imagined food consumption. We formulated this hypothesis because self-regulatory depletion has been shown to reduce habituation in other contexts (Wagner and Heatherton, 2013). We applied a 2 (depletion vs. non-depletion of self-regulatory resources) × 2 (*imagery item*: walnuts vs. coins) between-subjects design. We varied whether participants completed a task that depleted or did not deplete their self-regulatory resources. Furthermore, we asked participants to imagine either consuming food or putting a coin into a laundry machine. As the target food, we used walnuts in Study 2.

### MATERIAL AND METHODS

#### Participants

For Study 2, we recruited exclusively female participants via online forums, social networks, and message boards at the University of Vienna. We decided to include only women in the present study because the probability of observing an effect with a small sample size would be higher in a homogenous sample. In Study 1, men tended to eat more than women. Therefore, in Study 2, 90 females participated in the experiment. Four participants had to be excluded from the study because they failed the manipulation check. Four participants were excluded from the statistical analyses to preserve data homogeneity (cut-off > 2.5 SD of mean walnut consumption). Hence, data from 82 female participants were included in the statistical analyses. These participants had a mean age of 24.52 years (SD = 3.19) and a mean BMI of 21.38 kg/m^2^ (SD = 2.70). 11 were classified as overweight (BMI ≥ 25 kg/m^2^), one as obese (BMI ≥ 30 kg/m^2^), whereas all other participants displayed a normal BMI between 18.5 and 24.99 kg/m^2^. Most of the participants were undergraduate students in the nutritional sciences who volunteered to take part in the experiment.

Similar to Study 1, participants were deprived of food for 3 h and blind to the true purposes of the study, believing that they were participating in a taste test of different brands of walnuts. As compensation, every participant received a lottery ticket. As in Study 1, all ranges of BMI and age were included in the sample.

#### Study design

Study 2 was designed as a camouflaged taste test of walnuts, but in contrast to Study 1, the taste test was preceded by two interventions. First, a counting task with two different variations of difficulty was applied in order to induce a state of high and low self-regulatory depletion (Webb and Sheeran, 2003; Hagger and Chatzisarantis, 2013). Subsequently, the mental imagery paradigm was performed as the second task in the dual-task procedure either by asking participants to imagine that they were eating walnuts or, as a control, to imagine putting a 50¢ coin into a laundry machine with a given number of 18 repetitions. Participants were randomly assigned to a 2 (depletion vs. non-depletion of self-regulatory resources) × 2 (*imagery item*: walnuts vs. coins) between-subjects design using an online randomizer tool (Urbaniak and Plous, 2008). The amount of walnuts consumed was the main dependent variable in this experiment, and the questionnaire results of the taste tests were not analyzed further.

#### Procedure

Participants completed questionnaires with regard to their current hunger and overall liking of walnuts and stated their age, height, and body weight. Next a “test of mathematical abilities” was introduced, but it was in fact a counting task that was based on a test for assessing automatization difficulties in patients with dyslexia (Fawcett et al., 1996; Webb and Sheeran, 2003). The test was used to manipulate self-regulatory depletion in the present study. Participants in the high self-regulatory depletion condition were told to count backward from one thousand in multiples of seven while standing on only one leg. This procedure has been shown to evoke self-regulatory depletion in the past because participants need to resist the desire to quit this exercise due to their struggle to try not to lose their balance while engaging in a complicated counting task (Hagger and Chatzisarantis, 2013). Participants in the low self-regulatory depletion condition were instructed to count backward from 500 in multiples of five while standing on both legs, a task that was expected to require no self-control. After that, participants completed three manipulation check items to assess effort, difficulty, and fatigue (Webb and Sheeran, 2003) and rated their current mood by completing the short German version of the profile of mood states (Dalbert, 1992). Afterward, participants were seated in one of the classrooms of our facilities. We ensured that participants were not distracted in any way and seated them in an empty classroom. They were given detailed written instructions on the mental imagery task procedure (see Table S1). As in Study 1, we asked the participants to focus on sensory and textural characteristics of the imagined food item and on the eating experience. Participants in the control group were asked to focus on the sensory and textural characteristics of the imagined coin item and on the action (motoric) experience of throwing the coin in the laundry machine. To keep track of each repetition, we asked the participants to count each repetition on a checklist using pen and paper. We verified the checklists at the end of the experiment. Additionally, we encouraged the participants to spend at least 15 s for every repetition, but did not measure the cumulative time spent for the complete mental imagery task and therefore could not assess how much time each individual spent for the task.

Subsequently, participants engaged *ad libitum* in a taste test of different brands of walnuts, which were weighed before and after the experiment unbeknownst to the participants. Each participant was presented a total of 120 g of walnuts equally distributed in four identical bowls. Then participants completed the mental imagery manipulation check and the sub-scale for restrained eating from the Dutch Eating Behavior Questionnaire and the short version of the Barratt Impulsiveness Scale. Finally, participants were debriefed, thanked, and dismissed after receiving a lottery ticket as compensation.

### MEASURES

#### Manipulation check

To examine whether participants really performed the required mental imagery task, they were asked to indicate whether they conducted the task in the instructed way or not (“Did you really perform the task we asked you to?”).

#### Mood

Positive and negative mood states were examined. The short German version of the profile of mood states was used (Dalbert, 1992). After an initial question “How do you feel right at this moment?” participants rated 19 different items including grief (*n* = 3), desperation (*n* = 3), rage (*n* = 3), fatigue (*n* = 4), and positive mood (*n* = 6) on seven-point Likert scales ranging from 1 (*not at all*) to 7 (*very much*). Internal consistencies were α (*grief*) = 0.86, α (*rage*) = 0.77, α (*desperation*) = 0.81, α (*fatigue*) = 0.91, and α (*positive mood*) = 0.89.

#### Hunger status

To measure hunger before and after the experiment, a VAS with a length of 100 mm was used with four anchor points consisting of “not hungry at all,” “hungry,” “very hungry,” and “extremely hungry” (Stubbs et al., 2000). Liking of walnuts was assessed using the same VAS with three anchor points used in Study 1 for the gummy bears.

#### Task perceptions

We also measured whether self-regulatory depletion was successfully induced by the manipulation. Participants were instructed to rate the counting task on seven-point Likert scales ranging from 1 (*not at all*) to 7 (*very much*) according to whether the task was fatiguing, difficult, and required effort. The internal consistency in the current study was α (difficult, fatiguing, effortful) = 0.87 (Webb and Sheeran, 2003). High values indicate a strong self-regulatory depletion.

#### Trait impulsiveness

The short German version of the Barratt Impulsiveness Scale-15 was used to assess trait impulsiveness (Meule et al., 2011). This measure is commonly used to measure impulsive behavior as a trait. The Barratt Impulsiveness Scale-15 consists of three factors, which are non-planning, motor, and attentional impulsivity. Each factor consists of five items, which were rated on a 4-point scale ranging from 1 (*never*) to 4 (*always*). Statements for non-planning include “I plan tasks carefully”; for attention impulsivity, “I am restless during lectures or talks”; and for motor impulsivity, “I say things without thinking” (Spinella, 2007). The items were averaged into an overall impulsivity measure. High values indicate high trait impulsivity. Internal consistency in the present study was α (Barratt Impulsiveness Scale-15) = 0.78.

#### Restrained eating

The German translation of the sub-scale for restrained eating of the Dutch Eating Behavior Questionnaire was used (Van Strien et al., 1986). This scale consists of 10 items that target restrained eating with questions such as “When you have put on weight, do you eat less than you usually do?” or “Do you deliberately eat less in order to avoid becoming heavier?” These questions were answered on a 5-point Likert scale ranging from 1 (*never*) to 5 (*very often*). Internal consistency in the present study was α (*Dutch Eating Behavior Questionnaire*) = 0.91. High values indicate restrained eating.

#### Eating behavior

The amount of walnuts consumed as assessed by weighing the walnuts before and after the experiment with a standard scale to three decimals places served as the primary dependent variable.

#### Ethics statement

The experimental procedure was reviewed and approved by the University of Vienna Ethics Committee (*reference number*: 00065), and written informed consent was obtained from all participants before data collection. Participants were informed that they could withdraw their participation at any time during the experiment.

#### Statistical analysis

Results were considered significant at an α level of *p* ≤ 0.05 (results marked *ns* refer to* p*-values > 0.05).

### RESULTS AND DISCUSSION

#### Self-regulatory depletion induction

**FIGURE 2:**
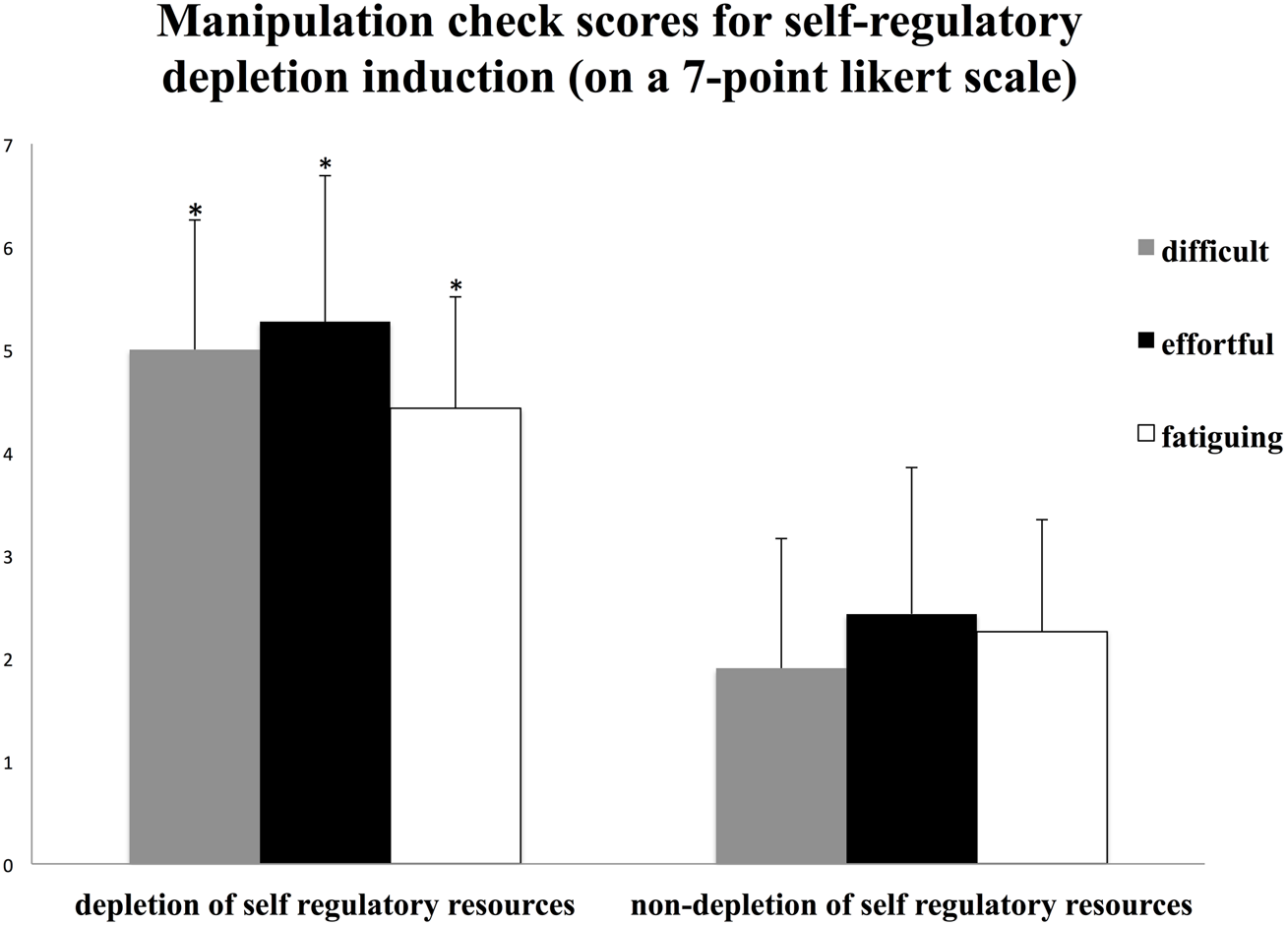
**Manipulation check scores for self-regulatory depletion induction (on 7-point Likert scale) displaying difficulty, effort and fatiguing as a function of resource depletion (depletion vs. non-depletion of self-regulatory resources).** Asterisk indicates *p* < 0.05.

Independent *t*-tests revealed significant differences in perceived difficulty, effort, and fatigue between the two ego-depletion conditions. In the depleted self-regulatory condition, participants rated the counting task as significantly more difficult, *t*(80) = 11.65, *p* < 0.001; effortful, *t*(80) = 7.87, *p* < 0.001; and fatiguing, *t*(80) = 12.50, *p* < 0.001, than participants in the non-depleted self-regulatory condition (**Figure 2**). This indicates a successful induction of self-regulatory depletion (Hagger and Chatzisarantis, 2013; Hagger et al., 2013). The self-regulatory depletion manipulation did not affect positive mood [*F*_(1,80)_ = -1.41, *ns*], grief [*F*_(1,80)_ = 0.85, *ns*], desperation [*F*_(1,80)_ = 0.24, *ns*], rage [*F*_(1,80)_ = 0.71, *ns*], or overall fatigue [*F*_(1,80)_ = 1.19, *ns*].

#### Eating behavior

We hypothesized that participants would eat a smaller amount of walnuts when they repeatedly imagined eating walnuts compared with imagining putting coins into a laundry machine. Furthermore, we expected this effect to be less prevalent in a state with depleted self-regulatory resources compared with a state with non-depleted resources. To test the hypotheses, we computed a 2 (depletion vs. non-depletion of self-regulatory resources) by 2 (imagery item: walnuts vs. coins) between-subjects ANOVA with walnut consumption as the dependent variable.

**FIGURE 3:**
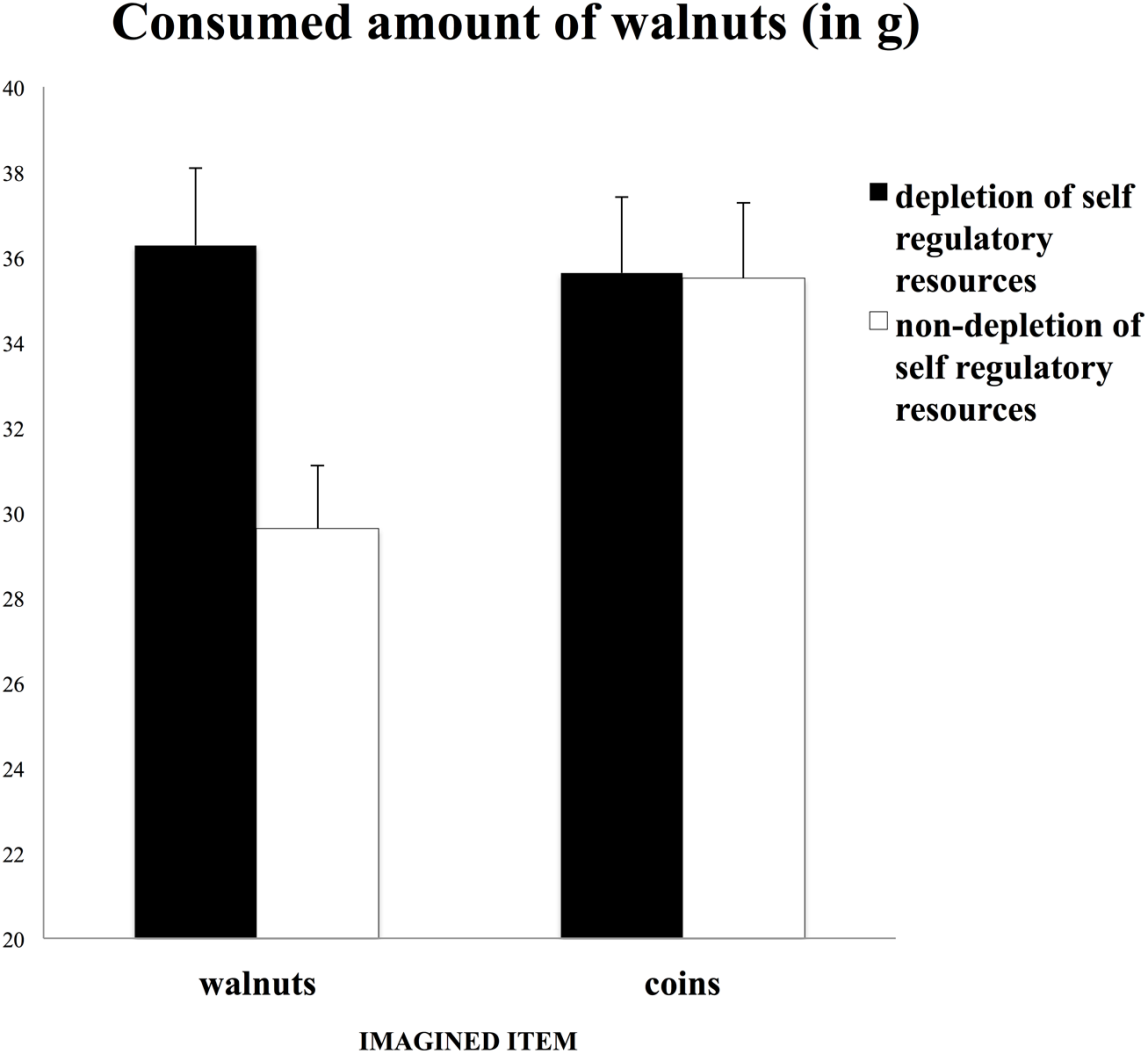
**Consumed amount of walnuts (in g) as a function of imagined item (walnuts vs. coins) and state of self-regulatory resources (depletion vs. non-depletion of self-regulatory resources).** Error bars indicate standard errors of the means (Study 2).

The results are depicted in **Figure 3**. In line with our hypothesis, participants in the non-depletion condition ate a smaller amount of walnuts when they imagined eating walnuts (*M* = 29.62 g, SD = 7.53 g) compared with when they imagined putting coins into a laundry machine (*M* = 35.49 g, SD = 7.53 g) [*F*_(1,39)_ = 6.26, *p* = 0.02, $\eta_{p}^{2}$ = 0.14]. By contrast, participants in the depletion condition did not differ in their intake of walnuts when they imagined eating walnuts (*M* = 36.29 g, SD = 9.77 g) compared with when they imagined putting coins into a laundry machine (*M* = 35.63 g, SD = 8.14 g), [*F*_(1,39)_ = 0.06, *p* = 0.82]. The interaction between self-regulatory depletion and the imaginary item was only marginally significant but had a medium effect size [*F*_(3,80)_ = 3.18, *p* = 0.08, $\eta_{p}^{2}$ = 0.04; see **Figure 3**]. The main effect of repeatedly imagining eating walnuts (*M* = 32.95 g, SD = 8.91 g) compared with repeatedly imagining putting coins into a laundry machine (*M* = 35.27 g, SD = 7.74 g) on the amount of walnuts consumed was not significant [*F*_(3,80)_ = 2.03, *p* = 0.16, $\eta_{p}^{2}$ = 0.03]. The main effect of the self-regulatory depletion manipulation on the amount of walnuts consumed was marginally significant. Participants ate more walnuts when self-regulatory resources were depleted (*M* = 37.89 g, SD = 11.26 g) compared with when they were not depleted (*M* = 33.26, SD = 9.36), [*F*_(3,80)_ = 3.43, *p* = 0.07, $\eta_{p}^{2}$ = 0.04].

A one-way analysis of covariance revealed that none of the following parameters showed effects on the amount of walnuts consumed: (i) BMI scores [*F*_(1,80)_ = 0.01,* p* = 0.93]; (ii) hunger prior to the experiment [*F*_(1,80)_ = 0.10, *p* = 0.92]; (iii) impulsivity scores [*F*_(1,80)_ = 0.03, *p* = 0.86]; (iv) restrained eating scores [*F*_(1,80)_ = 0.16, *p* = 0.69]; and (v) liking walnuts [*F*_(1,80)_ = 0.24, *p* = 0.63]. Liking walnuts did not change significantly from before (*M* = 82.61, SD = 24.65) to after the experiment was conducted (*M* = 84.38, SD = 23.70) [*F*_(1,80)_ = 1.91, *p* = 0.17]. There were no changes in hunger from before (*M =* 41.13, SD = 20.92) to after the mental imagery task (*M* = 27.03, SD = 20.51) across intervention groups [*F*_(1,82)_ = 0.15, *p* = 0.93].

To sum up, in Study 2, we found initial evidence that self-regulatory depletion can reduce habituation effects on the intake of food after imagining the consumption of the food.

## GENERAL DISCUSSION

Recent research has provided initial evidence that imagining the consumption of food leads to habituation effects that are similar to those that occur with the actual consumption of food (Morewedge et al., 2010). The objective of the present research was to replicate this effect with different kinds of foods and to test the hypothesis that self-regulatory depletion reduces habituation effects from the imagined consumption of food. The main reasoning for the latter was that habituation effects are basically memory effects that require cognitive resources. Therefore, the depletion of cognitive resources should reduce habituation effects that occur from imagining the consumption of food. The results of two studies showed that the habituation effect from food consumption is a stable phenomenon that occurs for different kinds of foods. Study 1 provided a replication of the initial habituation effects induced by mental imagery by Morewedge et al. (2010) and generalized these over another kind of food (gummy bears) as well as over different (smaller and larger) amount of imagery repetitions. In addition, the results of Study 2 provided initial evidence that the depletion of self-regulatory resources impedes habituation effects from imagining the consumption of food.

We replicated the findings of previous experiments using mental imagery (Morewedge et al., 2010) with two different food items (gummy bears and walnuts) with different optical and perceived health characteristics. The finding that habituation occurred with both types of food is in line with the assumption that habituation effects are independent of food characteristics (Epstein et al., 2009b). Furthermore, 18 and 36 repetitions showed similar effects on food intake (Study 1), indicating that 18 repetitions of mental imagery are enough to induce the habituation effect. Study 1 provided a replication of the initial habituation effects induced by mental imagery by Morewedge et al. (2010) and generalized these over another kind of food (gummy bears) as well as over different (smaller and larger) amount of imagery repetitions. We know from previous studies that three repetitions are not sufficient (Morewedge et al., 2010). Hence, the habituation-inducing threshold lies between 3 and 18 repetitions. This finding has implications for future research, because high numbers of repetitions can have side effects such as depletion or even impatience of participants and can lead to infeasibility of the mental imagery task. The finding that habituation effects occur on lower levels shows that it is possible to study the phenomenon with a decreased amount of repetitions. This implies that the phenomenon of habituation is limited in the extent, and that more repetitions do not necessarily lead to stronger habituation effects. Conducting 18 repetitions can be a time consuming task, thus future research should try to narrow down the threshold in which habituation to imagined foods occurs, using lower amounts of repetitions. A lower number of repetitions might lead to a more practicable approach using mental imagery and consequent habituation to reduce food intake.

At this point, we can only speculate why the different amount of imagery repetitions yielded a habituation effect of the same strength. Assumingly, the central process of imagery and habituation in the working memory might offer the answer. The working memory is a system with limited cognitive capacity (Cowan, 2004), and it is therefore possible that performing a vivid mental imagery task more than 18 times (36) might be overly taxing for the working memory (image vividness is related to capacity of cognitive resources (Bywaters et al., 2004)) in that the retrieval, maintenance, and refreshment of a repeated vivid mental imagery might have used all the available (working memory) resources (Kosslyn, 1996; Gunter and Bodner, 2008). As habituation also needs memory capacity to take place, we could assume that when the memory resources reached a capacity limit after a certain number of repetitions of the vivid mental imagery (we encouraged the participants to vividly imagine the food consumption), there were no more resources which could be used to strengthen the habituation effects even more over the next couple of repetitions.

To date, habituation effects of imagined food consumption have been found with M&M’s, cheese cubes (Morewedge et al., 2010), gummy bears (Study 1), and walnuts (Study 2). Similar to the studies by Morewedge et al. (2010), the results of the present studies showed that imagining the consumption of food does not reduce hunger or lead to a feeling of fullness.

Habituation effects after imagining food consumption do not represent demand effects because they deviate from the common expectation that thinking about food consumption increases appetite and hunger. In Study 1, we asked participants about their expectations and found that most of them expected an increase in consumption after imagining food consumption.

An interesting question is whether habituation effects from imagining food consumption follow the same rules as other habituation effects. Previous research has shown that habituation effects for example, those related to the perception of pictures or the consumption of food are reduced when individuals are distracted (Epstein et al., 2009b; Harris et al., 2009) or when their self-regulatory resources are depleted (Wagner and Heatherton, 2013). The finding from Study 2 that habituation was reduced when individuals’ self-regulatory resources were depleted implies that the habituation effects from visualization are based on processes that are similar to those involved in other forms of habituation.

It is difficult to explain the observed finding that the depletion of self-regulatory resources reduces the effects of imagined food consumption by reducing impulse control after self-regulatory depletion alone (Baumeister et al., 1998; Epstein et al., 2009b). First, walnuts are not a product that is related to strong impulses as chocolate and sweets are. Second, if there were strong impulses to eat walnuts, and if individuals needed self-regulatory resources to limit themselves when eating walnuts, the depletion of self-regulatory resources should have increased food intake in both imagination conditions (walnuts and coins) and not only in the condition in which walnut consumption was imagined.

From a different theoretical perspective, other promising studies have also examined the effects of thoughts on food, satiety, and how much such foods are liked (Papies et al., 2012; Redden and Galak, 2013; Larson et al., 2014). Papies et al. (2012) argue, for example, that spontaneous mental images can lead to a more abstract representation of food with a reduced focus on eating; this in turn reduces appetite, liking, and automatic approach responses to food. We cannot rule out the possibility that repetition effects also lead to a different representation of food. However, the mentioned stream of research did not study repetition effects as we did in the current study, and they did not predict differences between a single instance of imagining and the repeated imagination of food consumption.

A strength of the current research is that we measured actual food intake. Indeed, we observed a reduction in actual food intake ranging from 20 to 25% from repeatedly imagining food intake.

### LIMITATIONS

Although findings from both presented studies are intriguing, we want to mention that the interpretation of the results should be cautious because the interaction between self-regulatory depletion manipulation and the imaginary item was only marginally significant. The hypothesis that self-regulatory depletion reduces habituation effects after imagining the consumption of food is in line with models proposing that cognitive resources are needed for habituation effects to occur (Epstein et al., 2009b). Indeed, we suppose that the self-regulatory depletion task we applied in Study 2 slowed down the memory processes that are involved in habituation effects. However, at present, we cannot rule out the possibility that the self-regulatory depletion task reduced engagement in the imagination task or impeded attention allocation for the task (Friese et al., 2012; Inzlicht et al., 2014). Self-regulatory depletion might make it difficult for participants to imagine food consumption vividly, and one might speculate that a vivid imagination is a necessary precondition for habituation effects. In addition, it is possible that self-regulatory depletion amplifies processing at a lower level of brain processes but does not affect the memory processes that underlie habituation. Hence, on the basis of the present studies, we can conclude that self-regulatory depletion reduces the habituation effects of imagined food consumption.

But we can only speculate about the underlying processes. We would assume that the self-regulatory depletion decreased habituation effects in Study 2 by impeding the process of habituation. We assume that the self-regulatory task depleted cognitive resources (mainly memory) essential for habituation to take place and therefore habituation itself did not occur as a consequence. Apart from that, however, one might speculate that habituation is not only influenced by memory processes but also by other components of self-regulation leading to a reduced intake in participants with depleted self-regulatory resources. Beyond working memory, presumably disrupting self-regulation leads to a disruption of willpower resources, as well. Nevertheless, there is evidence that habituation enfolds slower in people who are allegedly weaker in self-regulation, such as people with obesity (Temple et al., 2007; Epstein et al., 2011), although cognitive resources are intact. Therefore, self-regulatory processes might influence habituation effects beyond just cognitive resources by additionally influencing resources of willpower. In fact, the depletion task used in Study 2 involved a component of extra self-control (balancing on one leg), which might have led to induce depletion of extra self-regulatory resources and thus those were responsible for reduced habituation. Future research might focus in more detail on the processes that underlie habituation effects after the imagination of food consumption and help to show which memory and brain processes are involved in such effects.

### CONCLUSION

The findings of this paper further elucidate how cognitive processes interfere with and shape eating behaviors. The results suggest that habituation after the repeated imagination of food consumption is a stable phenomenon that needs self-regulatory resources to occur.

## Conflict of Interest Statement

The authors declare that the research was conducted in the absence of any commercial or financial relationships that could be construed as a potential conflict of interest.
