# Supplementary material for: Mental imagery interventions reduce subsequent food intake only when self-regulatory resources are available
Source: Front Psychol. 2014 Nov 28;5:1391. doi: 10.3389/fpsyg.2014.01391 (PMC4246674; doi:10.3389/fpsyg.2014.01391)
Supplement: Supplementary file 1 [file Table_1.DOCX]

Table 1: Mental Imagery task instruction

**Study 1**

Mental Imagery induction - intervention with gummy bears

1. Positioning on chair as comfortable as possible keep your head straight.
2. Mental Imagery.
   1. close eyes.
   2. think of gummy bears in a bowl in front of you.
   3. grab one gummy bear, look at it and smell it.
   4. insert the gummy bear in your mouth an chew – feel the differences in texture and the salivation which emerge after chewing for a while.
   5. swallow chewed food and imagine the gummy bear going down your throat.
3. pause.
4. replicate task for given repetition.

Mental Imagery induction - control group

1. Positioning on chair as comfortable as possible, keep your head straight.
2. Mental Imagery task.
   1. close your eyes.
   2. think of a 50 ¢ coin in a bowl in front of you.
   3. grab the coin and take a look at it .
   4. put the 50 ¢ coin in a laundry machine.
   5. press reset to get back the coin.
3. pause.
4. replicate task for given repetition.

**Study 2**

Mental Imagery induction - intervention with walnuts

1. Positioning on chair as comfortable as possible keep your head straight.
2. Mental Imagery.
   1. close eyes.
   2. think of walnuts in a bowl in front of you.
   3. grab one walnut, look at it and smell it.
   4. insert one walnut in your mouth an chew – feel the differences in texture and the salivation which emerge after chewing for a while.
   5. swallow chewed walnut and imagine the food going down your throat.
3. pause.
4. replicate task for given repetition.

Mental Imagery induction - control group

1. Positioning on chair as comfortable as possible, keep your head straight.
2. Mental Imagery task.
   1. close your eyes.
   2. think of a 50 ¢ coin in a bowl in front of you.
   3. grab the coin and take a look at it .
   4. put the 50 ¢ coin in a laundry machine.
   5. press reset to get back the coin.
3. pause.
4. replicate task for given repetition
