# Supplementary material for: Mental imagery interventions reduce subsequent food intake only when self-regulatory resources are available
Source: Front Psychol. 2014 Nov 28;5:1391. doi: 10.3389/fpsyg.2014.01391 (PMC4246674; doi:10.3389/fpsyg.2014.01391)
Supplement: Supplementary file 2 [file Table_2.DOCX]

Table 2: Data summary, Studies 1 and 2

|  | Study 1 (n=95) | | | | Study 2 (n=82) | | | |
| --- | --- | --- | --- | --- | --- | --- | --- | --- |
| **Mental imagery task** | gummy bears  (18 rep) | gummy bears  (36 rep) | coins  (18 rep) | coins  (36 rep) | walnuts  (depletion) | walnuts  (no-depletion) | coins  (depletion) | coin  (no-depletion) |
| **consumed amount of food in gram** | 24.53 | 24.73 | 30.82 | 35.07 | 36.28 | 29.62 | 35.63 | 35.5 |
| **age in years** | 24.19 | 23.77 | 24.46 | 24.21 | 24.95 | 25.10 | 23.80 | 25.20 |
| **male** | 4 | 3 | 4 | 7 | 0 | 0 | 0 | 0 |
| **female** | 19 | 21 | 20 | 17 | 21 | 21 | 20 | 20 |
| **BMI in kg/m^2^** | 21.16 | 22.39 | 21.36 | 22.85 | 21.05 | 22.01 | 20.31 | 21.62 |
| **last meal intake in hours** | 3.13 | 3.01 | 3.75 | 4.5 | 4.91 | 5.24 | 4.15 | 4.68 |
| **hunger (pre-experimental)** | 26.90 | 30.18 | 35.21 | 39.33 | 41.91 | 36.48 | 47.63 | 38.70 |
| **hunger^ ^(post-experimental)** | 24.48 | 23.71 | 34.75 | 32.83 | 38.38 | 23.86 | 32.40 | 23.60 |
| **liking of target food (pre-experimental)** | 79.71 | 80.30 | 77.42 | 80.21 | 77.62 | 86.62 | 84.00 | 82.25 |
| **liking of target food^ ^(post-experimental)** | 76.22 | 77.42 | 76.67 | 79.83 | 76.14 | 92.33 | 86.05 | 83.00 |
| **fullness (pre-experimental)** | 49.57 | 56.23 | 48.92 | 43.46 | n.d | n.d | n.d | n.d |
| **fullness^ ^(post-experimental)** | 62.04 | 63.88 | 52.50 | 54.79 | n.d | n.d | n.d | n.d |
| **VVIQ** | 3.94 | 3.93 | 3.77 | 3.72 | n.d | n.d | n.d | n.d |
| **IDQ** | 3.46 | 3.25 | 3.26 | 3.05 | n.d | n.d | n.d | n.d |
| **RS** | 1.23 | 1.16 | 1.15 | 1.22 | n.d | n.d | n.d | n.d |
| **BIS-15** | n.d | n.d | n.d | n.d | 29.24 | 29.29 | 31.20 | 30.85 |
| **DEBQ** | n.d | n.d | n.d | n.d | 26.81 | 28.33 | 26.10 | 26.55 |

***Notes****.* BIS-15 = Barratt Impulsiveness Scale; BMI = Body Mass Index; DEBQ = Dutch Eating Behavior Questionnaire; IDQ = Individual Difference Questionnaire; n.d = no data; VVIQ = Vividness of Visual Imagery Questionnaire; RS = Restraint Scale
